# The significant coronary tortuosity and atherosclerotic coronary artery disease; What is the relation?

**DOI:** 10.15171/jcvtr.2018.36

**Published:** 2018-12-13

**Authors:** Mohsen Khosravani-Rudpishi, Adel Joharimoghadam, Elham Rayzan

**Affiliations:** ^1^Department of Cardiology, Science and Research branch, AJA University of Medical Sciences, Tehran, Iran; ^2^Universal Scientific Education and Research Network (USERN), Tehran, Iran

**Keywords:** Female, Coronary Angiography, Coronary Artery Disease, Diastole

## Abstract

***Introduction:*** Although coronary tortuosity is relatively common in coronary angiograms, there is much debate over the significance of this anatomical variation. So in this study the relation between significant coronary tortuosity (SCT) and coronary artery disease (CAD) was examined.

***Methods:*** The cross-sectional study included 737 patients (57% male) who were admitted to the hospital for a coronary angiography, based on their symptoms or non-invasive imaging. Coronary arteries defined as SCT are in the presence of either ≥3 consecutive curvatures of 90◦ to 180◦ or ≥2 consecutive curvatures of ≥180◦ measured at the end-diastole, in a major epicardial coronary artery ≥2 mm in diameter.

***Results:*** 29.17% of the patients had SCT of which females (64.7% vs. 34.1%, *P *< 0.001) and higher aged persons (62.9±8.4 vs. 57.8±10.7 years ± SD; *P * < 0.001) were significantly associated with SCT compared to non-SCT. Left anterior descending artery (LAD), left circumflex artery (LCX) and right coronary artery (RCA) with SCT in comparison to non-SCT, had lesser probability of CAD with stenosis severity of ≥50% (34.5% vs. 46.1%; *P * = 0.019 and 17.7% vs. 31.1%; *P * = 0.001 and 27.9% vs. 43.5%; *P * = 0.013 respectively) and also had significant lower Gensini scores (4.1±5.3 vs. 8.4±11.9; *P * = 0.011; 2.1±3.4 vs. 5.2±9.5; *P * = 0.01 and 1.2±1.9 vs. 5.03±8.9; *P * < 0.001 respectively) but higher TIMI frame count (15.7±5.3 vs. 11.9±4.6; *P * < 0.001 and 17.1±4.4 vs. 12.7±4.4; *P * < 0.001 and 15.2±3.9 vs. 11.6±4.8; *P * < 0.001 respectively).

***Conclusion:*** SCT is negatively correlated with CAD and there is a significant association between SCT and reduced coronary flow rate.

## Introduction

Tortuosity of the coronary arteries is a common finding during coronary artery catheterization, however, the importance of this phenomenon is not well-defined. Although aging, smoking, and hypertension have been shown as the probable risk factors of coronary tortuosity (CT), the main risk factors of CT have remained obscure.^1-3^ Significant coronary tortuosity (SCT) is a term of confliction and the proper definition is not globally accepted. Some researches define SCT as ≥3 consecutive curvatures of 45 degrees or more in at least one coronary bed,^1,4^ others consider 90 degrees curvatures as SCT.^3^ Furthermore, the impact of this phenomenon on cardiac events following coronary artery disease (CAD) has been less studied. It is hypothesized that SCT could be associated with increased coronary artery calcium score (CAC score),^1^ myocardial perfusion defect and chest pain in normal coronary artery patients^5^ and even spontaneous coronary artery dissection.^6^

Meanwhile little is known about the association of coronary atherosclerosis and SCT. Some studies declare a positive correlation between subclinical atherosclerosis and CT,^1^ although some others are in the opposition.^4^ This study has been designated in order to clarify this correlation.

## Materials and Methods

All the patients underwent a coronary angiography based on the clinical indications, from December 2016 to December 2017 (2016-1017) in the AJA university hospital, Tehran, Iran, were included and the informed consents were collected. Among 1043 patients, 737 were included in this study. The patients with a past history of coronary artery bypass graft (CABG), percutaneous coronary intervention (PCI), and patients presented by acute ST elevation myocardial infarction were excluded. Also, the patients with chronic total occlusion (CTO) in the angiograms, without acceptable anterograde or retrograde run-off were omitted. All coronary angiograms were done by the conventional methods with femoral or radial artery approach and filmed at 15 frames per second by a Siemens radiographic unit (Siemens Healthcare, Germany). Angiograms were reviewed by two expert cardiologist who were both blind to the study. The first one localized SCT and the other one evaluated the severity, site and length of stenosis, calcification, ectasia or aneurysm. The visually assessed luminal coronary obstructions of ≥50% were considered as CAD. The TIMI flow (0, 1, 2 and 3) and the TIMI frame count were calculated. The first frame used for TIMI frame counting was the first frame in which the contrast material fully enters the coronary artery while 1) a column of nearly full or fully concentrated dye extends across the entire width of the origin of the artery, 2) the dye touches both borders of the origin of the artery and 3) the anterograde motion of the dye exists. The last frame count is the one in which the dye first enters the end-point branch of the targeted artery. Due to the longer length of left anterior descending artery (LAD), TIMI frame count of LAD was divided by 1.7 and corrected TIMI frame count was calculated.^7^ In the original study on the calculation methods of TIMI frame count,^7^ all angiograms were filmed at 30 frames per second, therefore we multiplied TIMI frame counts by 2.

The Gensini score was used for quantifying coronary stenosis. In this method the severity of coronary stenosis is classified as up to 25%, 50%, 75%, 90%, 99% and 100%. By giving a score of 1 for up to 25 percent obstruction and doubling the score as the severity of the obstruction progresses. Accordingly, 2 is given for 50%, 4 for 75%, 8 for 90%, 16 for 99% and 32 for a total occlusion. Each segment of the coronary artery is given a multiplying factor from 0.5 to 5, based on the functional significance of the area supplied by that segment. Finally, the Gensini score is the sum of calculated scores.^8^

**Figure 1:**
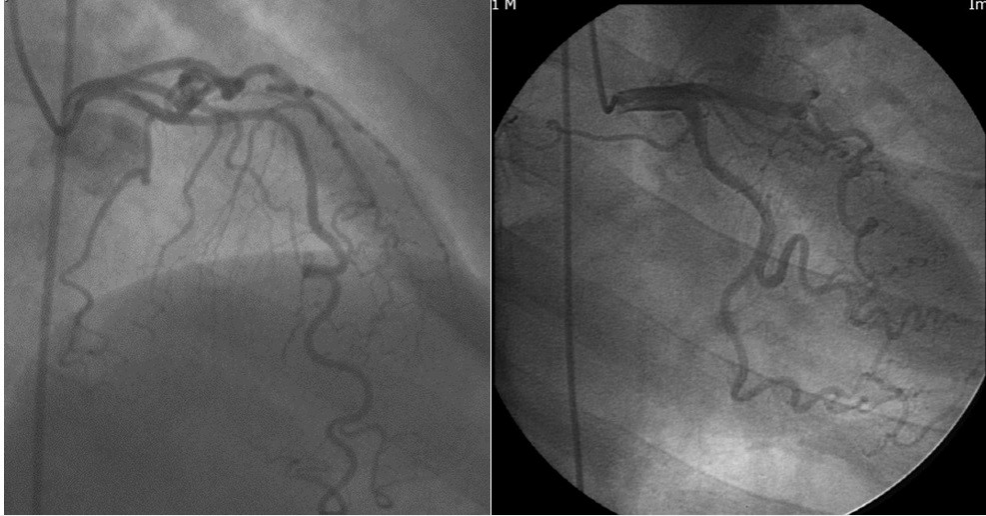
Significant coronary tortuosity (SCT).

Mild CT was described as ≥3 consecutive curvatures of 45° to 90° in a major epicardial coronary artery, or ≥3 consecutive curvatures of 90° to 180° in an artery, <2 mm in diameter.^6^ Moderate CT was defined by the presence of ≥3 consecutive curvatures of 90° to 180° measured at the end-diastole in a major epicardial coronary artery, ≥2 mm in diameter.^6,9^ Severe CT was defined as ≥2 consecutive curvatures of ≥180° in a major epicardial coronary artery, ≥2 mm in diameter.^6,10^ SCT defined as either moderate or severe CT (Figure 1).

### Statistical analysis

Data analysis was done by IBM SPSS software (version 19, SPSS, Inc.). Continuous variables were presented as mean ± SD. The normally distributed continuous variables were compared between groups using the Student t-test and in case of not-normally distributed variables, Mann-Whitney U test was applied. Categorical variables were examined with chi-square or Fisher exact test, as appropriate. The *P* value less than 0.05 (2 sided) was considered statistically significant. Multivariate logistic regression analysis was performed in order to adjust demographic parameters which directly affecting the coronary tortuosity.

## Results

This study included 737 patients with a predominantly male population (57 %) and the CAD rate was 61.7%. Nearly 50 % of patients had CT which was categorized as mild (20.8 %), moderate (17.1 %) and severe (12.1 %) (Table 1). Left circumflex artery (LCX) was the most prevalent coronary artery involved with SCT. The pattern of SCT in coronary arteries is shown in Table 2. Being female (64.7% vs. 34.1%, *P*<0.001) and of higher age (62.9±8.4 vs. 57.8±10.7 years ± SD; *P*<0.001) were significantly associated with SCT but SCT did not show any significant correlations with the other risk factors of atherosclerosis (Table 3).

**Table 1:** Severity of coronary tortuosity

**Artery**	**Tortuosity**			
	**No**	**Mild**	**Moderate**	**Severe**
LAD	520 (70.6%)	98 (13.3%)	102 (13.8%)	17 (2.3%)
LCX	399 (54.1%)	174 (23.6%)	83 (11.3%)	81 (11%)
RCA	609 (82.6%)	60 (8.1%)	51 (6.9%)	17 (2.3%)
Overally	369 (50.1%)	153 (20.8%)	126 (17.1%)	89 (12.1%)

LAD, left anterior descending artery; LCX, left circumflex artery; RCA, right coronary artery.

**Table 2:** Pattern of significant coronary tortuosity

**Artery**	**All patients with SCT (n=215)**	**Male** **(n=76)**	** Female** ** (n=139)**
LAD	31 (14.4%)	11 (14.5%)	20 (14.4%)
LCX	73 (34.0%)	34 (44.7%)	39 (28.1%)
RCA	6 (2.8%)	0 (0%)	6 (4.3%)
LAD & LCX	42 (19.5%)	9 (11.8%)	33 (23.7%)
LAD & RCA	14 (6.5%)	11 (14.5%)	3 (2.2%)
LCX & RCA	17 (7.9%)	5 (6.6%)	12 (8.6%)
LAD & LCX & RCA	32 (14.9%)	6 (7.9%)	26 (18.7%)
Total	215 (100%)	76 (100%)	139 (100%)

LAD, left anterior descending artery; LCX, left circumflex artery; RCA, right coronary artery; SCT, significant coronary tortuosity

**Table 3:** Baseline characteristics and significant coronary tortuosity

**Variable**	**Total (n=737)**	**Non-SCT (n=522)**	**SCT (n=215)**	***P*** ** value**
Age (y)	59.3 ± 10.3	57.8 ± 10.7	62.9 ± 8.4	<0.001
Body mass index, kg/m^2^	26.5 ± 2.1	26.4 ± 2.2	26.8 ± 1.9	0.063
Female gender	317 (43%)	178 (34.1%)	139 (64.7%)	<0.001
Diabetes	82 (11.1%)	59 (11.3%)	23 (10.7%)	0.812
Hypertension	326 (44.2%)	231 (44.3%)	95 (44.2%)	0.987
Dyslipidemia	243 (33%)	172 (33%)	71 (33%)	0.985
Smoking	108 (14.7%)	72 (13.8%)	36 (16.7%)	0.303
Aspirin	175 (23.7%)	123 (23.6%)	59 (27.4%)	0.267
Beta blocker	180 (24.4%)	120 (23%)	60 (27.9%)	0.158
ACEi or ARBs	198 (26.9%)	148 (28.4%)	50 (23.3%)	0.156
Statins	123 (16.6%)	84 (16.1%)	41 (19%)	0.327

ACEi, angiotensin converting enzyme inhibitor; ARBs, angiotensin II receptor blockers; SCT, significant coronary tortuosity.

**Figure 2:**
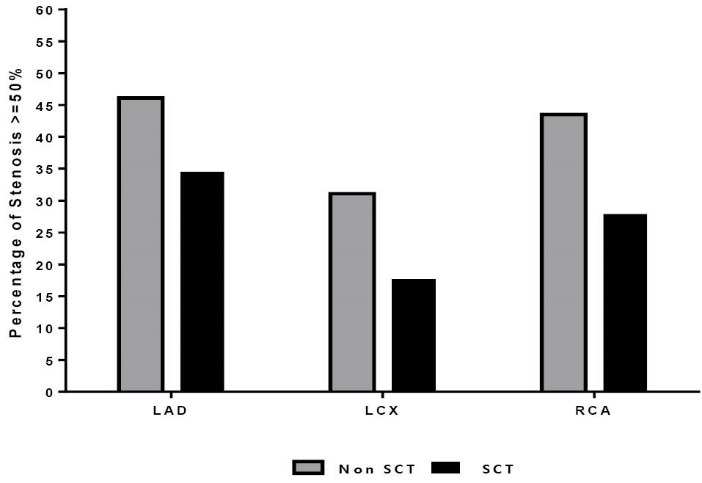
Stenosis ≥50% and significant coronary tortuosity (SCT).

**Figure 3:**
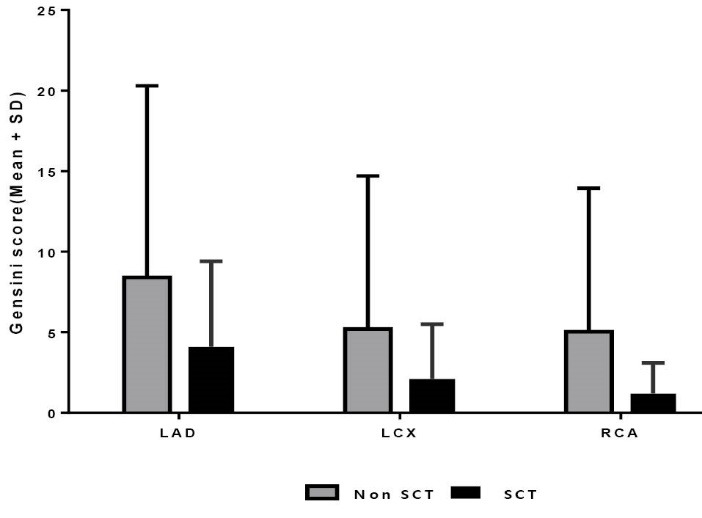
Gensini score and significant coronary tortuosity (SCT)

All three coronary arteries (LAD, LCX and RCA) with SCT in comparison to non-SCT had a lesser probability of CAD with stenosis severity of ≥50% (34.5% vs. 46.1%; *P*=0.019 and 17.7% vs. 31.1%; *P*=0.001 and 27.9% vs. 43.5%; *P=0.013* respectively) (Figure 2) and also had significant lower Gensini scores (4.1±5.3 vs. 8.4±11.9; *P*=0.011; 2.1±3.4 vs. 5.2±9.5; *P*=0.01 and 1.2±1.9 vs. 5.03±8.9; *P*<0.001 respectively) (Figure 3).

Slower flow rate in coronaries was also more prevalent in SCT as estimated by TIMI frame count, which was higher in all three coronary beds (LAD, LCX & RCA) with SCT vs. non-SCT (15.7±5.3 vs. 11.9±4.6; *P*<0.001 and 17.1±4.4 vs. 12.7±4.4; *P*<0.001 and 15.2±3.9 vs. 11.6±4.8; *P*<0.001 respectively). The angiographic characteristics related to SCT are shown in Table 4.

**Table 4:** Angiographic characteristics and significant coronary tortuosity

**Factor**	**LAD**			**LCX**			**RCA**		
	** Non-SCT**	**SCT**	** P **	**Non-SCT **	**SCT**	**P **	**Non-SCT**	**SCT**	**P**
Calcification	76 (12.3%)	10 (8.4%)	0.226	43 (7.5%)	2 (1.2%)	0.001	66 (9.9%)	2 (2.9%)	0.060
Ectasia	60 (9.7%)	5 (4.2%)	0.052	26 (4.5%)	8 (4.9%)	0.855	83 (12.4%)	2 (2.9%)	0.016
Stenosis ≥ 50%	285 (46.1%)	41 (34.5%)	0.019	178 (31.1%)	29 (17.7%)	0.001	291 (43.5%)	19 (27.9%)	0.013
Length of stenosis Discrete	88 (36.8%)	25 (61%)	0.002	79 (55.6%)	22 (75.9%)	0.123	94 (38.4%)	12 (63.2%)	0.093
Tubular	49 (20.5%)	10 (24.4%)		41 (28.9%)	4 (13.8%)		104 (42.4%)	6 (31.6%)	
Diffuse	102 (42.7%)	6 (14.6%)		22 (15.5%)	3 (10.3%)		47 (19.2%)	1 (5.3%)	
TIMI frame count	11.9±4.6	15.7±5.3	0.000	12.7±4.4	17.1±4.4	<0.001	11.6±4.8	15.2±3.9	<0.001
Gensini score	8.4±11.9	4.1±5.3	0.011	5.2±9.5	2.1±3.4	0.010	5.03±8.9	1.2±1.9	< 0.001

LAD, left anterior descending artery; LCX, left circumflex artery; RCA, right coronary artery; SCT, significant coronary tortuosity.

After adjusting the odds ratio of SCT patients for age and gender, the observed results of ≥50% for coronary stenosis and Gensini scores still remained persistent (Table 5).

**Table 5:** Adjusted Odds ratio of having significant coronary tortuosity

**Factor**	** LAD **		**LCX**		** RCA**	
	AOR (95% CI)*	**P **	AOR (95% CI)*	**P **	AOR (95% CI)*	** P**
Stenosis ≥ 50%	0.57 (0.37-0.88)	0.011	0.72 (0.44-1.17)	0.182	0.48 (0.27-0.85)	0.011
Gensini score	0.95 (0.92-0.98)	0.001	0.9 (0.89-0.98)	0.007	0.82 (0.72-0.92)	0.001

*Adjusted odds ratio for Age and Gender LAD, left anterior descending artery; LCX, left circumflex artery; RCA, right coronary artery.

## Discussion

Although CT is relatively a common finding in coronary angiographies, there is much debate over the significance of this anatomical variation. It was found that SCT is negatively correlated with atherosclerosis in both genders. SCT was analyzed for each coronary artery separately in order to omit the biased results, because CAD and SCT can occur simultaneously in a single patient’s different coronary arteries.

This is the first study which evaluates the CT in detailed and comprehensively in order to find the correlation between SCT and atherosclerotic CAD. It was found that SCT is related to less severe coronary stenosis and less coronary atherosclerotic burdens with a trend toward less diffused coronary artery stenosis. Findings reported by Li et al^11^ and also supported by Joseph Chiha et al^4^ showed that the Gensini score (10.4 vs. 15.5, *P* = 0.02) and the Extent scores (12.4 vs. 19.1, *P*=0.03) were significantly lower in females with CT in comparison to the non-CT group. Esfahani et al^12^ showed that the mean of Gensini index was 53 ± 43.9 in the tortuous group and 80 ± 75 in the nontortuous group (*P* = 0.003)

Some studiesdefined severe CT as the presence of ≥3 bends of ≥45° deviation in the vessels direction.^1,4^ This was considered as mild CT in this study. Tortuosity was classified in three groups as mild, moderate and severe, moreover, considering mild tortuosity and no tortuosity patients in a non-SCT group versus a SCT group (moderate and severe CT).

Mohammad El Tahlawi et al^1^ studied the CT angiograms of 83 patients and divided them into two groups based on the presence of coronary tortuosity. They considered tortuosity as ≥3 consecutive bends ≥45^◦^ in at least one coronary bed. There was a direct correlation between the groups’ CAC scores and the coronary tortuosity score (*P* < 0.05). As CAC score is a strong indicator of atherosclerosis, the mentioned results are somehow different from the ones found. It might be due to the different methods used for calculating the coronary tortuosity. Another study in which the degrees of CT were not differentiated in the groups of mild, moderate and severe, showed a significant lower Gensini score only in female with tortuosity.^4^

This study showed that SCT is positively associated with female gender and aging. These findings are supported by several studies.^4,12,13^

It was also shown that SCT is associated with increased TIMI frame count, further emphasizing the slower flow rate in comparison to non-SCT patients. Obviously the higher the TIMI frame count, the lower the coronary flow rate. Therefore, the slow flow coronaries have a higher TIMI frame count. Some studies concluded that the tortuosity of femoral or carotid arteries are associated with atherosclerotic disease but the results obtained were different for coronary arteries.^14-16^ As the coronary blood flow is predominantly diastolic^17^ and as SCT resulted in slower flow rate in coronary arteries, the shearing stress might be lessened on the endothelial cells in comparison to high systolic flow and shearing stress in non-coronary vessels, a hypothesis which should be investigated by more hemodynamic and invasive studies.

CT can result in a reduction in coronary perfusion pressure, leading to ischemia in the absence of luminal narrowing. It has also been suggested that CT causes an alteration in blood flow and reduces the coronary artery pressure distal to the tortuous segment.^18^ In another study using numerical simulation, Li et al^19^ showed that CT can result in decreased coronary blood pressure regarding its severity. Therefore, severe CT may cause myocardial ischemia and angina pectoris.

Some studies have shown that the patients with angina pectoris without significant coronary obstruction, have reversible myocardial perfusion defects based on MPI^5^ and dobutamine stress echocardiography^3^, Hence, the hypothesis of microvascular disease derived angina pectoris was suggested.

In the presenting study, patients underwent a coronary angiography following the positive results of non-invasive imaging or positive cardiac symptoms. Those patients whom identified as non-significant CAD, had higher probability of SCT and higher TIMI frame counts which may emphasize the possibility of microvascular dysfunction. This issue needs more precise studies by means of CMR or PET scans for investigation of absolute flow reserve of SCT vs. non-SCT patients in order to detect the possible association with microvascular dysfunction.

## Limitations

This study was a single-center cross-sectional study of patients undergone coronary angiography, therefore the comparability to the general population was limited. Utilizing CMR and PET scan for evaluating microvascular dysfunctions was not possible due to limited access and financial problems.

## Ethical approval

The proposal was not assessed by the ethical committee at the time of approval due to the cross-sectional nature observation.

## Competing interests

All authors declare no competing financial interests exist.
